# The Lègami/Legàmi Service—An Experience of Psychological Intervention in Maternal and Child Care during COVID-19

**DOI:** 10.3390/pediatric13010021

**Published:** 2021-03-22

**Authors:** Giovanna Perricone, Ilenia Rotolo, Viviana Beninati, Nicolò Billeci, Valeria Ilarda, Concetta Polizzi

**Affiliations:** 1 Società Italiana di Psicologia Pediatrica (S.I.P.Ped), 90144 Palermo, Italy; giovanna.perricone@unipa.it (G.P.); ilerotolo@gmail.com (I.R.); vbeninati@libero.it (V.B.); nico.billeci@gmail.com (N.B.); valeria.ilarda@gmail.com (V.I.); 2 Department of Psychology, Educational Science and Human Movement, University of Palermo, 90128 Palermo, Italy

**Keywords:** psychological support, COVID-19, needs, difficulties, resources

## Abstract

This study provides a descriptive analysis of the Lègami/Legàmi service, a free psychological support service in maternal and childcare, offered through the internet and by telephone that was initiated by the Italian Society of Pediatric Psychology (S.I.P.Ped.) during the COVID-19 medical emergency as an act of solidarity, first independently, and then in collaboration with the Italian Ministry of Health. This paper presents findings related to the “universe” of people who called the toll-free service, from the sociocultural characteristics of the users to the information collected by the professionals during the psychological pathways until human satisfaction was achieved. We provide a retrospective description of an experience that took place between April and June 2020, and which involved users of the maternal-infant area calling from the whole Italy. (1) Methods: The aims of this study were to investigate the configuration of the indicators identified and to detect the possible correlations between them in the sample. There were 193 users who took advantage of the Service, 160 of whom continued beyond the reception service; it is this group that we report the findings from here. The tool used was a form reporting access to care and interventions, and the resulting data underwent a content analysis and the indicators were subject to non-parametric statistical analysis to analyze differences and relationships. (2) Results: There were many correlations among the indicators that revealed a high prevalence of calls due to personal motivations and requests for support, which later allowed users to gain a greater understanding of the underlying problems they were facing. The professionals running the service noticed a prevalence of weaknesses attributable to the negative emotions of its users, alongside a presence of cognitive and relational resources. The professionals’ interventions, which can be characterized by a prevalence of social support, psychological rehabilitation, and psychoeducation, achieved outcomes of redefining users’ relationships with themselves and others. All of the service’s users have expressed a high level of satisfaction with it. (3) Discussion: Our results revealed the protective and transformative effects of the service for its users and the underlying importance of having an easily accessible psychological support system in place during emergencies, like the recent pandemic. In conditions like these, the great value of a remote support service should be noted, and despite its limitations, assures its own efficacy when a medical emergency precludes closer in-person forms of psychological assistance.

## 1. Background

Here, we present a descriptive study of the psychological support offered by the Lègami/Legàmi service initiated by the Società Italiana di Psicologia Pediatrica (S.I.P.Ped) during the national lockdown in response to the COVID-19 pandemic. This period has presented real risks for everyone and a sudden and unexpected destructuralization of developmental balances, leading to greater needs in terms of psychological support [1,2,3,4,5].

We present and discuss data collected from the users of the service, which was organized by the S.I.P.Ped as an act of solidarity, first independently, then in collaboration with the Italian Ministry of Health. The latter invited all of the societies registered in the specific list of the “Scientific Societies and Technic-scientific Associations of Healthcare Occupations” to manage the second level of the Service’s psychological support via a free phone number.

Lègami/Legàmi is a community proximal remote service (accessible through online platforms and by telephone) [6], which was established to meet the current need of its prospective users, providing an immediate answer, and reaching each subject exactly in their context of life (home or work environment). Thanks to the online tools, it was possible to intervene in a user’s daily life, in their so-called “natural environment” as the principal setting of their suffering, providing an opportunity to extend the psychological intervention to their family.

Online and telephone support were chosen as the exclusive channels of help because of the isolation measures faced during the national lockdown. It should be noted that the Lègami/Legàmi Service conforms with the CNOP’s (National Council of Psychologists Order) Guidelines published in 2017 within the document “Digitalization of the profession and the psychological intervention mediated by web”, and to the latest directions provided by CNOP (2020) about the remote psychological intervention in support of the population during the COVID-19 emergency.

Even before the pandemic, remote psychological consultation had a methodological validity in maternal-infant healthcare [7], and it has been used by internationally known helplines (e.g., NSPCC, Childline, Allo119). This kind of consultation allows professionals to adapt their interventions to new and upcoming needs by responding to the user’s needs in a direct and immediate manner.

The Italian Department of Health, referring to the communication of the European Committee COM (2008) “Telemedicine for the benefit of patients, of healthcare systems and society”, in March 2014 issued the national guidelines regarding telemedicine, with a reference to tele-psychology as part of the national strategy in the field of healthcare, with attention to the population’s health needs and proximal assistance methods. 

Several studies have shown the benefits of telemedicine, highlighting the volume of users it can reach, the reduction in waiting times, and its applicability under various risk conditions, although the need to deepen and broaden the research to evaluate the effectiveness of online psychological support has almost always been emphasized. Indeed, the usefulness of this kind of psychological consultation has frequently been relied on in emergency situations such as disasters, suicide attempts, trauma, or with cancer patients support [8,9,10], those with neuromuscular disorders [11], and in mental health disorders [12,13]. Subjects who underwent online mindfulness interventions experienced positive outcomes in terms of emotional regulation, anxiety management, and stress reduction support [14]. Online cognitive behavioral therapy interventions are also widely used with post-secondary students to reduce emotional distress symptoms [15]; other similar interventions are employed to reduce perinatal anxiety and depression [16]. 

Nowadays, psychological consultation features as a fundamental medium for the whole community, not just those with specific vulnerability conditions; during the pandemic, tele-psychology helped address issues for users of all ages and with varied needs.

The model of the Lègami/Legàmi service’s psychological intervention considered the participation of users as a narrative experience [17,18], functioning to give meaning to the historic moment being lived and to the personal need of support. In this regard, it was possible to develop a mental organization of a personal biography [19], in which to rebuild one’s inner world in relation to the historic time of the COVID-19 pandemic and the time that followed. In this personal biography, it was possible to develop other models representing the person’s relationships, affections, and place in the community. The professionals used a psychological intervention model based on listening [20,21], accompaniment [22], reception [23], mentoring, anticipation of events [24], and adaptation [25].

Listening was carried out through a kind of “consultation setting”, which, through considerations made out loud and detecting techniques, enabled the user to see alternative ways of managing events and emotions, in a path in which the accompaniment represented the possibility of “understanding together with the user”, while the mentoring made it possible to “see” the user and implement the alternatives identified. The strength of the intervention model was the duplicity and ambivalence of the “anticipation of events” [24]—by following the user’s dysfunctional anticipation of catastrophic hypotheses, the professional could guide a rewriting of such anticipations through social support. Following this model, the professional practice included social support [26,27] and the buffering effect it can have [28,29]; psychological, educational, and psychosocial rehabilitation [30,31,32,33,34]; and clinical, observational, experiential, and psychodiagnostic methods. Furthermore, the psychological intervention offered the user to recognize and distinguish between the request made, the type of support indicated, and the implicit need [35].

The intervention pathway took into consideration the importance of the reason for calling, as the most explicit and explanatory cause of the reasons for the call, which had been sent from the first level to the second level (“I have called because…”); the type of support, which was well-defined and highlighted by the colleagues in the reception (“I am calling because understood that…”) [20,21,22,23]; and the type of implicit request outlined after the first meeting with the professional (“I am here for…”).

Within the management of the Legami/Legàmi service, the reception had a fundamental guidance function for access to the service, often dispelling the users’ misguided expectation of an “immediate telephone therapy”. The interview at the reception was important to promote the users’ trust and willingness to rely on the personnel; indeed, the reception represented the first link with the service, as well as the possibility to join a real support organization. 

In the intervention model, developing the user’s request and identifying the complex psychological functioning, including weaknesses and resources, defined the boundaries of the users’ inner space. This provided a focus to attempt to “fix” (in the sense of reconnecting the meanings) the representations of the world and one’s own place in the world, the “before” and the “here and now” of COVID-19, returning to the user their possibility of agency with respect to the emergency. This led to functional outcomes of being better able to cope with the critical event, in terms of emotions, representations, behaviors, and relationships with the self and others, even if the weaknesses and related negative emotions had originated before COVID-19. 

Thus, in a moment marked by fears, anxieties and loss of certainty, the service was for many people the only relational space available to tell of their experience of suffering. Indeed, participants trusted professionals and entrusted themselves to them, and when it was proposed, they accepted the referral to follow-ups or to other professionals of the service, or to some discussion groups. This trust was also expressed in welcoming the suggestion, in some cases, to turn to specialized regional services (e.g., Child and Adolescent Neuropsychiatry).

## 2. Materials and Methods

We investigated the evolution and contextualization of the pandemic emergency on a social and psychosocial level by setting the following goals:

- Investigate the configuration of the indicators identified within the same factor.
- Evaluate the differences between indicators of the same factor.
- Examine possible correlations between the indicators identified.
- Test for the presence of significant differences between indicators.

### 2.1. Participants

**Figure 1:**
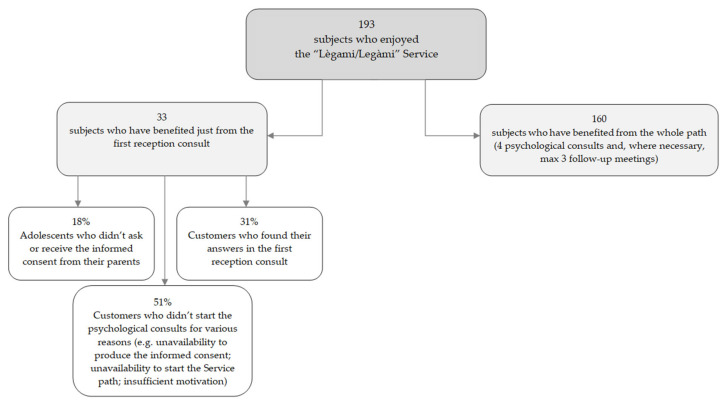
Distribution of the sample.

A total of 193 users sought help from the Lègami/Legàmi service. They were mainly referred by the societies managing the first level service within a larger project run by the Ministry of Health. Among these, 160 users (83%) benefitted from the whole path provided by the service (reception and psychological interviews), while the remaining 33 users (88% female) only benefitted from the interviews at reception (Figure 1). Some of these users were adolescents who did not continue the path because they could not and/or had no intention to ask for their parents’ informed consent; the remainder were adults, mainly parents, who often received the support they required during the reception interview (Figure 1). 

Users that followed the entire treatment pathway (n = 160; 79% female) had an average of 3.8 interviews (SD = 1.3), while 16% also had follow-up interviews (maximum of 3), either individually or in a group. Just over half (52%) of users asked for psychological consultation via phone, while the remainder preferred remote online platforms (Skype, Zoom, etc.) or WhatsApp. Most of the users in the sample were between 36 and 46 years old (41%). The distribution of the geographic origin indicated that 36% of the users called from northern Italy, 24% from central regions, 9% from southern Italy, and 31% from the islands, predominantly the Sicilian region (29%), perhaps because some groups (teachers and parents) had been referred directly by regional bodies. Finally, the sample consisted equally of married/cohabiting (48%) and single people, mainly with a medium-high socio-cultural level (Table 1). 

### 2.2. Service Organization and Criteria for Access 

The service included a team of 36 psychologists who conducted the psychological interventions, a management group composed of a supervisor, coordinator, two psychologists responsible for the telephone reception part of the process, and an operator who collected the questionnaires and carried out data analysis. 

The service provided two fundamental steps: The reception and the psychological consultation. The reception pathway consisted of some operational steps—welcoming the user, presenting the service, accepting and listening to the user’s reasons for calling, and assisting the subject about the fruition of the psychological support. Then, the following consultation pathway was conducted by the team of psychotherapists, who provided four psychological interviews, which might close the support intervention or lead to a referral to regional healthcare services, taking account of the user’s mental health condition at the end of the psychological consultation.

The Lègami/Legàmi service used some indicators shared at the ministerial level with other scientific societies to guide the action of listening, which led to the following specific criteria defining the psychological care:

- Not to activate psychotherapy paths in the traditional sense.
- To carry out a limited number of interviews.
- Foresee the possibility of referral to groups and/or other professionals of the service and/or regional healthcare services.
- Activate the process of developing the user’s request, from an explicit level to an implicit level. This list is defined under Article 5 of Italian Law number 24 of 8 March 2017 and under the Italian Ministerial Decree of 2 August 2017—Resolution of the Ministry of Health, DGPROF n.0053321-P-06/11/2018; Societies and Associations also being part of the Advisory Body set up by the National Board of Italian Psychologists (Decision of the CNOP 21 June 2019).
- Look at the complexity of the user’s functioning, including weaknesses and resources.
- Take account of models of psychological intervention, both of a clinical and psychosocial nature.

### 2.3. Tools and Procedures

We collected data using a form concerning topics such as access to care and intervention. It was filled in by the psychologists and divided into three topics, each of which had specific factors: (1) A description of the user (personal data, reason for calling, user’s request, developing the user’s request, resources and weaknesses); (2) the professional’s choices, with factors including the intervention model adopted, potential referral to internal or external services; and (3) information related to relapses and thus the professional’s considerations, underlining any potential changes observed in the user. 

The research project has been approved by the Ethical Committee of the Società Italiana di Psicologia Pediatrica.

### 2.4. Data Analysis

The data collected in the forms underwent a content analysis, achieving specific indicators for each factor. The indicators were as follows:

- *Reason for calling*: Personal reasons, reasons concerning the reference system (e.g., the couple, children, etc.) and reasons concerning the role held (e.g., work role).
- *User’s request*: Listening, orientation, support, and therapy.
- *Developing the user’s request*: Awareness of problematic focal point, self-awareness, giving sense and meaning/finding alternatives, and strengthening.
- *User’s weaknesses*: Red flags for psychopathology, negative emotionality/mourning and loss/dysfunctional defenses, vulnerability in the relationship with the self and with others/lack of boundaries and weak self-regulation/relational difficulties, weaknesses, and vulnerabilities of the reference systems.
- *User’s resources*: Cognitive, relational/social, emotional/motivational/spiritual resources, and resources coming from training/personal paths.
- *Referral:* External referral (regional services) and referral within the Lègami/Legàmi service (groups, follow-up);
- *Intervention model*: Social support, psychological rehabilitation, educational rehabilitation, psychosocial rehabilitation, buffering hypothesis, and psychoeducation.
- *Professional’s considerations*: Redefining the relationship with the self and with others, discomfort in the relationship with the self and with others, showing hidden emotions and expressing them, and the need for continuous support.

Besides undergoing descriptive analysis, the data were also subject to non-parametric statistical analysis to assess differences and correlations among variables. Analyses were carried out using SPSS-IBM (New York, NY, USA) v.23. The descriptive analysis (mean ± SD) enabled us to examine the frequency distribution of the indicators. The Friedman test was used for the analysis of variance by ranks for k > 2 dependent/correlated samples in order to verify the presence of significant differences between indicators of the same factor. This analysis was carried out to determine which indicators were prevalent within the supported sample. We used Kruskal–Wallis tests to investigate differences in indicators according to user group membership, to evaluate the importance of socioeconomic attributes in determining the detected indicators. Finally, correlations among indicators were tested using Spearman’s non-parametric correlation coefficient (r_s_).

## 3. Results 

The three data topics and their respective indicators identified through the specific form of access to care and intervention are reported with reference to differences in importance among response indicators of the same considered topic (Table 2) and potential differences between indicators on the basis of the independent variables of the sample (Table 3 and Table 4). First, the “reason for calling” was identified as the most explicit form of need. As shown in Table 2, “personal reasons” were of significant importance; in terms of socioeconomic and geographic attributes (age, socio-cultural level, marital status, region), these were especially mentioned by widowers and single people, but also by very young people (10–18 years old) and by middle-aged and older people (58–69) (see Table 3). Users between 36 and 57 years old, who were often separated/divorced or widowers, mainly called for reasons linked to their children’s problems. It is interesting to note that adults under 50, especially employed women, often teachers, mainly called for reasons linked to their role and function (Table 3).

As for the “request for support explicitly expressed by the user” factor, the indicator of the request for support to manage emotional and relational issues was most prevalent (Table 1). This was mainly expressed by people from northern and central-northern regions, working as managers or retired, and those with a basic qualification (Table 3). The following factors in terms of relevance were the presence of “requests to listen” to the suffering condition, mainly among the elderly (75–85) and young adults (19–24 and 25–35 years). Finally, remarkably few “requests of orientation” were expressed, such as requests for information and suggestions, especially by adults (36–46) (see Table 3). 

Referring to the transformative value of the psychological intervention, a crucial passage is found in “developing the user’s request” as a transformation of the opening request for support into the identification of one’s deepest needs. This seems to have guided users firstly towards greater awareness of the problematic focal point for which help was being asked (Table 3), most prevalent among individuals with a medium–low level of education, and towards greater self-awareness, especially among unmarried women. Finally, the “need for strengthening” was most prevalent among retired and graduated people (Table 3).

In line with the need for emotional and relational support highlighted above, the main vulnerabilities pointed out by professionals (Table 2) included a pervasive negative emotion, both internalized and externalized, mainly present among students, and a vulnerability in the relationship with the self and others, mainly present among separated/divorced people (Table 3). Afterwards, red flags for psychopathology were identified as another kind of weakness/vulnerability, especially among pensioners (70–85 years), teenagers (14–18 years), and lonely people. Another critical area that emerged was regarding the vulnerability of the reference systems/social vulnerability (Table 2), most prevalent among users from some northern regions (Table 3).

The intervention of the service was characterized by finding both weaknesses and resources, among which the cognitive and relational/social resources were most relevant, followed by emotional/motivational resources and those linked to personal training (Table 2), mostly found among employees and freelancers (Table 4). With regard to the professional’s choices and the “referral” factor, we found that most of the referrals internal to the service concerned preadolescents (10–13 years old) and young people (19–24 years old), and a significantly higher number of referrals, both internal (e.g., follow-up groups) and external (e.g., services of mental health), concerned male users. 

Data concerning the “professional’s considerations” were related to the possible changes made by users due to the psychological intervention. Our results show that “redefining the relationship with the self and with others” was an outcome highlighted often by elderly people and adolescents, as well as by single people and widowers, and among those with lower-level secondary education (Table 4). “Showing hidden emotions and expressing them” was an outcome highlighted by many user groups. To a lesser extent, a persisting condition of “discomfort in the relationship with the self and with others” could be found among users (Table 2). All of the outcomes of change were derived from the activation of a psychological intervention mainly based on social support and psychological rehabilitation, especially with adults/elderly people (58–69 years old), and psychoeducation with young adults (25–35 years old) (Table 3 and Table 4). 

The data revealed significant correlations between vulnerabilities of users and personal resources. Notably, there was a significant relationship between having cognitive resources and presenting red flags for psychopathology or negative emotionality (rho = 0.019, *p* < 0.01; rho= 0.26, *p*= 0.001). We also found that the more the user presented weaknesses in the relationship with the self and with others, the fewer emotional (rho = −0.16, *p* = 0.04) and training resources (rho = −0.18, *p* = 0.02) they presented. However, data revealed a positive correlation between weaknesses in the relationship with the self and with others and relational resources (rho = 0.20, *p* = 0.01).

We detected significant correlations between “red flags for psychopathology” and a request for support mainly involving “personal reasons” (rho = 0.43, *p* < 0.01). Conversely, red flags for psychopathology were negatively correlated with reasons for calling linked to the reference systems (rho = −0.15, *p* = 0.04) and roles and functions (rho = −0.24, *p* < 0.01). Negative emotionality/mourning and loss/dysfunctional defenses were significantly positively associated with users who called for personal reasons (rho = 0.30, *p* < 0.01). The data showed positive correlations between red flags for psychopathology and referrals to the internal service (rho = 0.21, *p* < 0.01), and between negative emotionality and referrals internal to the service, especially follow-up interviews or groups (rho = 0.17, *p* = 0.02). Negative emotion was positively correlated with positive “professional’s considerations”, related to redefining of the relationship with the self and with others (rho = 0.16, *p* = 0.03), and with other less positive considerations such as discomfort in the relationship with the self and with others (rho = 0.17, *p* = 0.02) and showing hidden emotions and expressing them (rho = 0.22, *p* < 0.01). The results showed further correlations between user’s resources and reasons for calling: Users who owned more training resources were less likely to have called for personal needs (rho = −0.18, *p* = 0.01), but the reason for them calling was especially linked to roles and functions (rho = 0.31, *p* <0.01). Likewise, a positive correlation was identified between the cognitive resources of users and professional’s considerations about redefining of the relationship with the self and with others (rho = 0.22, *p* < 0.01).

## 4. Discussion and Conclusions

These results enable us to make some observations both on the psychological functioning of the users of the service and on its transformative and protective impacts. With regard to the stressful conditions being experienced by users who called the service, it should be noted that users who called due to personal problems were most often single people, for whom loneliness might have accentuated the suffering linked to the isolation, very young people (10–18 years old), and middle-aged and older people (58–69 years old). These findings confirm the scientific evidence that very young people and adults over 50 tend to request social support as the main coping mechanism, much more so than middle-aged people [36]. On the other hand, users between 36 and 57 years old mainly called for reasons linked to their children’s problems, and the fact that they were often separated, divorced, or widowers shows that during this pandemic, some of the most complex conditions were experienced by lone parents (Table 3). It is interesting to note that adults under 50 years old, especially employed women, often teachers, mainly called for reasons linked to their role and function (Table 3), a reason for calling that was positively associated with the presence of training resources and the professional self—many teachers asked for support in terms of orientation since they had plunged into deep crisis about how to manage the educational relationship. 

The pandemic and lockdown that followed have generated real risk of emergency conditions among people, which are often related to a sense of loneliness that is not necessarily related to there being an absence of other people around them. This has generated a huge need for listening and support in the face of their conditions, as shown by users’ questions addressed to the service’s professionals. In considering the intensity of such needs, the nearly even distribution of requests for support addressed to the service from the northern regions, central regions, and islands of Italy is particularly interesting. This underlines the “universal” fear of COVID-19, regardless of being in an area of high risk of contracting the virus or not, and emphasizes the state of anxiety caused by the lockdown [1,2,3,4,5]. 

Another interesting finding regards the clear prevalence of female users of the service. Indeed, calls often arrived from mothers worried about their children or their ability to manage their parental role. On the other hand, several studies highlight that the pandemic has often put in crisis the parental competence, also directing child neglect outcome [37,38,39,40,41]. In this regard, it is worth mentioning how mothers, as compared to fathers, are characterized by an innate physiological inclination to activate immediate responses of care for a child in difficulty or who is suffering [42,43]. It also seems that women tend to face difficulties through a request of social support, showing their feelings and communicating their difficulties more than men do [44]. Although many parents were calling for their children, the service then became an opportunity for the parents themselves. It is also worth mentioning the low level of direct participation in the service by children and adolescents, which may also be due to the greater difficulty in expressing a need verbally, instead expressing needs by symptomatology or dysfunctional behaviors. However, the “child/adolescent” focus has always been a priority for the Lègami/Legàmi service, based on the principles and constructs of Pediatric Psychology. Therefore, wherever possible, the professionals become directly involved with the child/adolescent via the parent or consider the parent as a force in the field of the developmental emergency experienced by the child [45].

Despite the low number of adolescents in this study, it should be noted that findings highlighted a significant vulnerability in the emotional self-regulation among very young people [46]. In particular, considering this weakness, it is believed that an important role was played by forced isolation, in a phase of social development characterized by continuously trying to be in contact with a group of peers outside of the family environment. These concerns led the Italian Higher Institute of Health to develop specific indications for the psychological support of minors during the pandemic [47]. The prevalence of weaknesses in terms of negative emotionality confirms the consideration about the incredible impact that the COVID-19 emergency has had on the emotional balance of people, determining a variety of maladaptive reactions [48,49], and strongly jeopardizing many people’s mental health [50]. Indeed, we wonder how many more people have turned to private and public services for mental health since the lockdown. It is interesting to highlight that, during the emergency, being well-equipped on a cognitive level was not always a protective factor. On the contrary, this may have led to attitudes of hyper-control and the constant search for information about contagions, mortality, etc., which must have exacerbated the discomfort and anxiety related to the virus. However, having cognitive resources is a relevant mediating factor concerning a possible transformation in the relationship with the self and with others.

Further considerations are suggested by our findings related to “developing the user’s request”, and about the transformation of the opening request for support into a greater awareness of the problematic focal point for which help was being asked and the need for strengthening. 

These data allow us to reflect on the role played by the service in the psychological but also psychoeducational rehabilitation as a pathway to understanding problems. One final remark needs to be made about developing the user’s request to give new meaning to the problems presented. An increased sense of agency and the development of a certain metacognitive mastery were highlighted, meant as a greater control and awareness of one’s thoughts and beliefs about the problems they experienced.

With regard to the outcomes of the service’s interventions and its methodological approach, some relapses should be noted in terms of cognitive coping and creative adaptation among users who benefitted from the support. Indeed, the service represented an incredible opportunity to express negative emotional states, but also to experience many positive emotional aspects that had been repressed. These outcomes were promoted by psychological interventions based on social support, psychological rehabilitation, and psychoeducation. The model of social support may have been particularly useful, such as some studies highlighted [51,52], because the service had to address problems that arose or were exacerbated during a moment of widespread social isolation [51]. At the same time, it showed its worth in answering the questions that many users were asking regarding the management of problems linked to the reference system (children, partner, etc.). Furthermore, psychological rehabilitation was found to be fundamental to addressing the needs linked to a clear prevalence of negative emotionality. Psychoeducation may also have played an important role in helping face the new challenges presented in daily life by the national lockdown.

To conclude, this study has helped us to highlight the importance of psychological intervention under the conditions of a health emergency via online support, which can foster wider participation. The study also reminds us that the need for psychological support is among the primary needs to be guaranteed, in line with the many years of work by the National Council of Psychologists to recognize psychological assistance for the Essential Levels of Assistance [53,54]. 

Methodologies including tele-consultation, tele-cooperation, and online psychotherapy and follow-up, seem underline potential effectiveness of tele-psychology; however, a major effort must be made to define specific guidelines and recommendation for professionals [55,56,57,58,59]. Moreover, this type of intervention could be employed in different cultures and various contexts, can help to reduce the risk of drop-out and build a strong therapeutic alliance, especially with pre-adolescents and adolescents, and with different conditions such as chronic disease or disability [60,61]. There is also a necessity to create pathways of online support specifically built to target pre-adolescents and adolescents [62,63]. Indeed, this study features a specific limitation due to the loss of a small number of participants (n = 33) who dropped out after the reception interview for various reasons. In particular, some adolescents (18%) dropped out before starting the consultation because they chose not to ask for the informed consent of their parents in fear of a potential negative reaction from their parents. To rectify this gap, in the future the service, as a permanent Community Proximal Service going beyond the pandemic, will develop a new reception interview aimed at strengthening adolescents’ motivations to begin the consultation, overcoming their fears. Finally, another limitation of the study is that it was set up as an explorative research study but did not use standardized tools to evaluate the users’ psychological conditions before and after the intervention, so there are a lack of data to generalize our findings to wider groups.

## Figures and Tables

**Table 1:** Characteristics of the sample (N = 160).

Variables	
**Age ranges (%)**	
10–13	6
14–18	9
19–24	6
25–35	12
36–46	41
47–57	18
58–69	6
70–85	2
**Gender (%)**	
Male	21
Female	79
**Region of origin (%)**	
Valle d’Aosta	0
Lombardia	15
Emilia Romagna	6
Toscana	3
Veneto	3
Trentino Alto Adige	2
Friuli Venezia Giulia	2
Piemonte	4
Liguria	1
Umbria	3
Abruzzo	1
Lazio	8
Marche	2
Molise	1
Campania	9
Puglia	6
Basilicata	0
Calabria	3
Sicilia	29
Sardegna	3
**Educational qualification (%)**	
Primary school diploma	11
Middle school diploma	16
Secondary school diploma	42
Post-secondary degree	31
**Profession (%)**	
Employee	39
Freelance worker	11
Manager	2
Temporary collaborator	6
Unemployed	18
Student	22
Pensioner	2
**Marital status (%)**	
Unmarried	21
Bachelor	12
Married	48
Divorcee	18
Widower	1

**Table 2:** Differences between the indicators of each detected variable (Friedman Test) (N = 160).

Variables	Mean (SD)	Friedman Test
**Reason for calling**		
Personal reasons	0.50 (0.5)	χ^2^ = 12.62 gl = 2 *p* = < 0.002
Reasons concerning the reference system	0.33 (0.4)	
Reasons concerning the role held	0.27 (0.4)	
**User’s request**		
Listening	0.10 (0.3)	χ^2^ = 164.66 gl = 3 *p* = < 0.001
Orientation	0.30 (0.4)	
Support	0.70 (0.4)	
Therapy	0.03 (0.1)	
**Developing the user’s request**		
Awareness of the problematic focal point	0.64 (0.5)	χ^2^ = 85.25 gl = 3 *p* = < 0.001
Self-awareness	0.44 (0.5)	
Giving sense and meaning/Find alternatives	0.28 (0.5)	
Strengthening	0.20 (0.8)	
**User’s weaknesses**		
Red flags for psychopathology	0.40 (0.7)	χ^2^ = 81.14 gl = 3 *p* = < 0.001
Negative emotionality/mourning and loss/Dysfunctional defenses	1.48 (1)	
Vulnerability in the relationship with the self and with others	1.18 (6)	
Weaknesses and vulnerabilities of the reference systems	0.52 (0.7)	
**User’s resources**		
Cognitive resources	0.86 (0.9)	χ^2^ = 94.06 gl = 3 *p* = < 0.001
Relational/social resources	0.85 (0.8)	
Emotional/motivational/spiritual resources	0.56 (0.7)	
Resources coming from training/personal paths	0.12 (0.3)	
**Intervention model**		
Social support	0.47 (0.5)	χ^2^ = 152.78 gl = 6 *p* = < 0.001
Psychological rehabilitation	0.31 (0.4)	
Educational rehabilitation	0.13 (0.3)	
Psychosocial rehabilitation	0.05 (0.2)	
Buffering hypothesis	0.12 (0.3)	
Psychoeducation	0.28 (0.4)	
Other models	0.01 (0.1)	
**Referral**		
External referral (regional services)	0.28 (0.4)	χ^2^ = 1.88 gl = 2 *p* = < 0.390
Referral within the Lègami/Legàmi Service (follow-up, groups)	0.34 (0.5)	
None	0.37 (0.5)	
**Professional’s considerations**		
Redefining of the relationship with the self and with others	1.08 (1)	χ^2^ = 95.43 gl = 3 *p* = < 0.001
Discomfort in the relationship with the self and with others	0.70 (1)	
Showing hidden emotions and expressing them	0.12 (0.3)	
Need for continuous support	0.30 (0.4)	

**Table 3:** Differences between the indicators of the detected variables (reason for calling, user’s request, developing the request for support, user’s weaknesses) and the independent variables (age, gender, region of origin, educational qualification, profession, marital status) (Kruskal–Wallis test) (N = 160).

Variables	Reason for Calling			User’s Request				Developing the User’s				User’s Weaknesses			
	Personal Reasons	Reasons Concerning the Reference System	Reasons Concerning the Role Held	Listening	Orientation	Support	Therapy	Awareness of the Problematic Focal Point	Self-Awareness	Giving Sense and Meaning/ Find Alternative	Strength-Ening	Red Flags for Psychopa-Thology	Negative Emotionality/Mourning and Loss/ Dysfunctional Defenses	Vulnerabi-Lity in the Relationship with the Self and with Others	Weaknesses and Vulnerabi-Lities of the Reference Systems
**Age ranges**	Mean (SD)			Mean (SD)				Mean (SD)				Mean (SD)			
10–13	1(0)	0 (0)	0 (0)	0 (0)	0 (0)	0.88 (0.3)	0 (0)	0.44 (0.5)	0.33 (0.5)	0.33 (0.5)	0.11 (0.3)	0.44 (1)	2.44 (0.2)	0.44 (0.5)	0.33 (0.5)
14–18	1.13 (0.5)	0 (0)	0 (0)	0 (0)	0.06 (0.2)	0.86 (0.3)	0.13 (0.3)	0.60 (1)	0.40 (0.5)	0.20 (0.4)	0.06 (0.2)	1.06 (1)	1.86 (1)	0.46 (0.6)	0.53 (0.9)
19–24	0.90 (0.3)	0.20 (0.4)	0 (0)	0.20 (0.4)	0.30 (0.4)	0.70 (0.4)	0.10 (0.3)	0.50 (0.5)	0.70 (0.4)	0.20 (0.4)	0 (0)	0.50 (0.7)	2.20 (1.8)	0.80 (1.3)	0.50 (0.8)
25–35	0.50 (0.5)	0.35 (0.4)	0.30 (0.4)	0.25 (0.4)	0.25 (0.4)	0.80 (0.4)	0 (0)	0.75 (0.4)	0.40 (0.5)	0.15 (0.3)	0 (0)	0.45 (0.8)	1.55 (1)	0.70 (0.8)	0.60 (0.8)
36–46	0.27 (0.4)	0.36 (0.4)	0.46 (0.5)	0.09 (0.2)	0.44 (0.5)	0.56 (0.4)	0.04 (0.2)	0.61 (0.4)	0.53 (0.6)	0.24 (0.4)	0.41 (1)	0.15 (0.4)	1.24 (1.2)	1.75 (10.5)	0.46 (0.6)
47–57	0.27 (0.4)	0.62 (0.4)	0.24 (0.4)	0.03 (0.1)	0.34 (0.4)	0.75 (0.4)	0 (0)	0.75 (0.5)	0.24 (0.4)	0.55 (0.7)	0.03 (0.1)	0.34 (0.7)	1.10 (1.4)	0.75 (0.9)	0.62 (0.7)
58–69	0.88 (0.3)	0.11 (0.3)	0.11 (0.3)	0.11 (0.3)	0.11 (0.3)	0.77 (0.4)	0 (0)	0.55 (0.5)	0.44 (0.5)	0.22 (0.4)	0.22 (0.4)	0.66 (1)	2.11 (1.4)	1.11 (1.6)	0.33 (0.5)
70–85	0.66 (0.5)	0.33 (0.5)	0 (0)	0.66 (0.5)	0 (0)	0.66 (0.5)	0 (0)	1 (0)	0.33 (0.5)	0.33 (0.5)	0 (0)	1.30 (1)	0.66 (0.5)	0 (0)	1.66 (2)
**Kruskal Wallis Test**	χ^2^ = 65.53; ***p* = < 0.001**	χ^2^ = 34.72; ***p* = < 0.001**	χ^2^ = 28.55; ***p* = < 0.001**	χ^2^ = 16.74; ***p* = < 0.019**	χ^2^ = 19.02; ***p* = < 0.008**	χ^2^ = 11.29; *p* = < 0.126	χ^2^ = 7.25; *p* = < 0.403	χ^2^ = 8.68; *p* = < 0.276	χ^2^ = 8.19; *p* = < 0.316	χ^2^ = 7.25; *p* = < 0.403	χ^2^ = 10.22; *p* = < 0.176	χ^2^ = 25.36; ***p* = < 0.001**	χ^2^ = 12.50; *p* = < 0.085	χ^2^ = 6.83; *p* = < 0.446	χ^2^ = 3.47; *p* = < 0.838
**Gender**															
Male	0.61 (0.4)	0.32 (0.4)	0.05 (0.2)	0.14 (0.3)	0.23 (0.4)	0.76 (0.4)	0.05 (0.2)	0.67 (0.7)	0.41 (0.4)	0.35 (0.5)	0.02 (0.1)	0.50 (0.8)	1.35 (1.4)	0.67 (0.8)	0.61 (0.7)
Female	0.47 (0.5)	0.33 (0.4)	0.33 (0.4)	0.09 (0.2)	0.32 (0.4)	0.68 (0.4)	0.03 (0.1)	0.63 (0.4)	0.45 (0.5)	0.26 (0.4)	0.24 (0.9)	0.37 (0.7)	1.51 (1.4)	1.23 (7.5)	0.50 (0.7)
**Kruskal Wallis Test**	χ^2^ = 2.90; *p* = < 0.08	χ^2^ = 0.58; *p* = < 0.445	χ^2^ = 10.06; ***p* = < 0.002**	χ^2^ = 1.10; *p* = < 0.292	χ^2^ = 1.62; *p* = < 0.202	χ^2^ = 0.43; *p* = < 0.512	χ^2^ = 0.72; *p* = < 0.394	χ^2^ = 0.06; *p* = < 0.795	χ^2^ = 0.03; *p* = < 0.858	χ^2^ = 0.42; *p* = < 0.514	χ^2^ = 2.42; *p* = < 0.120	χ^2^ = 0.37; *p* = < 0.539	χ^2^ = 0.32; *p* = < 0.567	χ^2^ = 1.19; *p* = < 0.275	χ^2^ = 0.86; *p* = < 0.353
**Region of Origin**															
Lombardia	0.70 (0.6)	0.41 (0.5)	0.08 (0.2)	0 (0)	0.16 (0.3)	0.91 (0.2)	0.04 (0.2)	0.83 (0.8)	0.33 (0.4)	0.37 (0.6)	0 (0)	.0.33 (0.7)	1.25 (1.3)	0.41 (0.6)	0.66 (0.7)
Emilia Romagna	0.60 (0.5)	0.30 (0.4)	0.20 (0.4)	0.20 (0.4)	0.10 (0.3)	0.80 (0.4)	0 (0)	0.40 (0.5)	0.30 (0.4)	0.50 (0.5)	0 (0)	0.60 (0.5)	1.50 (1.7)	8.90 (26.7)	1 (0.6)
Toscana	0.60 (0.5)	0.40 (0.5)	0 (0)	0.20 (0.4)	0.80 (0.4)	0.40 (0.5)	0 (0)	1.2 (0.4)	0.60 (0.5)	0.20 (0.4)	0 (0)	0.60 (0.5)	0.60 (0.8)	0.60 (0.8)	0.20 (0.4)
Veneto	0.40 (0.5)	0.60 (0.5)	0 (0)	0.40 (0.5)	0.40 (0.5)	0.60 (0.5)	0 (0)	0.60 (0.5)	0.60 (0.5)	0 (0)	0 (0)	0.60 (0.8)	2 (2.1)	0.60 (0.8)	0.60 (0.8)
Trentino Alto Adige	0.66 (0.5)	0.33 (0.5)	0.33 (0.5)	0 (0)	0 (0)	1 (0)	0 (0)	1 (0)	0 (0)	0 (0)	0 (0)	0 (0)	1.33 (1.5)	0.66 (0.5)	0.33 (0.5)
Friuli Venezia Giulia	0.66 (0.5)	0.33 (0.5)	0 (0)	0.33 (0.5)	0 (0)	1 (0)	0 (0)	1 (0)	0.33 (0.5)	0 (0)	0 (0)	1.33 (1.5)	2 (0)	1.66 (1.1)	2.66 (0.5)
Piemonte	0.57 (0.5)	0.28 (0.4)	0 (0)	0 (0)	0.28 (0.4)	0.57 (0.5)	0 (0)	0.28 (0.4)	0.57 (0.5)	0.28 (0.4)	0 (0)	0.28 (0.4)	1.71 (1.9)	0.57 (0.7)	0.28 (0.4)
Liguria	0 (0)	1 (0)	1 (0)	0 (0)	0 (0)	1 (0)	0 (0)	1 (0)	1 (0)	1 (0)	1 (0)	1 (0)	1 (0)	0 (0)	2 (0)
Umbria	0.60 (0.5)	0.60 (0.5)	0 (0)	0.20 (0.4)	0 (0)	1 (0)	0 (0)	0.80 (0.4)	0.20 (0.4)	0 (0)	0 (0)	0.60 (1.3)	1 (1.2)	0.80 (0.8)	0.60 (0.5)
Abruzzo	0 (0)	0 (0)	1 (0)	0 (0)	1 (0)	1 (0)	0 (0)	1 (0)	0 (0)	2 (0)	0 (0)	0 (0)	0 (0)	4 (0)	1 (0)
Lazio	0.84 (0.3)	0.30 (0.4)	0.15 (0.3)	0.15 (0.3)	0.15 (0.3)	0.76 (0.4)	0.15 (0.3)	0.61 (0.5)	0.46 (0.5)	0.23 (0.4)	0 (0)	0.53 (0.8)	1.53 (1.2)	0.30 (0.4)	0.76 (1.1)
Marche	1 (0)	0 (0)	0.33 (0.5)	0.66 (0.5)	0 (0)	1 (0)	0 (0)	1 (0)	0.66 (0.5)	0.66 (0.5)	0 (0)	0.66 (0.5)	1 (19	0.66 (0.5)	0 (0)
Molise	1 (0)	0 (0)	1 (0)	0 (0)	0 (0)	1 (0)	0 (0)	1 (0)	1 (0)	0 (0)	0 (0)	1 (0)	0 (0)	2 (0)	1 (0)
Campania	0.60 (0.5)	0.33 (0.4)	0.13 (0.3)	0 (0)	0.26 (0.4)	0.73 (0.4)	0.13 (0.3)	0.60 (0.5)	0.53 (0.6)	0.06 (0.2)	0. 13 (0.3)	0.33 (0.7)	1.80 (1.4)	0.46 (0.7)	0.26 (0.5)
Puglia	0.77 (0.4)	0.11 (0.3)	0.11 (0.3)	0.11 (0.3)	0 (0)	0.88 (0.3)	0 (0)	0.44 (0.5)	0.55 (0.5)	0.22 (0.4)	0.11 (0.3)	0.55 (0.7)	1.11 (1.2)	0.88 (0.7)	0.22 (0.4)
Calabria	0.25 (0.5)	0.75 (0.5)	0 (0)	0 (0)	0.50 (0.5)	0.50 (0.5)	0 (0)	0.75 (0.5)	0 (0)	0.25 (0.5)	0 (0)	0 (0)	2.25 (3.2)	0 (0)	0 (0)
Sicilia	0.17 (0.3)	0.23 (0.4)	0.65 (0.4)	0.06 (0.2)	0.54 (0.5)	0.47(0.5)	0.02 (0.1)	0.54 (0.5)	0.50 (0.6)	0.34 (0.5)	0.60 (1)	0.26 (0.7)	1.58 (1.4)	0.65 (1.1)	0.43 (0.5)
Sardegna	0.40 (0.5)	0.60 (0.5)	0 (0)	0.40 (0.5)	0.40 (0.5)	0.60 (0.5)	0 (0)	0.60 (0.5)	0.40 (0.5)	0.20 (0.4)	0 (0)	0.20 (0.4)	1.80 (1.4)	0.40 (0.5)	0 (0)
**Kruskal Wallis Test**	χ^2^ = 39.23; ***p* = < 0.002**	χ^2^ = 17.21; *p* = < 0.440	χ^2^ = 61.72; ***p* = < 0.001**	χ^2^ = 30.08; ***p* = < 0.026**	χ^2^ = 37.85; ***p* = < 0.003**	χ^2^ = 29.54; ***p* = < 0.030**	χ^2^ = 11.36; *p* = < 0.837	χ^2^ = 21.60; *p* = < 0.200	χ^2^ = 13.76; *p* = < 0.683	χ^2^ = 22.36; *p* = < 0.171	χ^2^ = 31.87; ***p* = < 0.016**	χ^2^ = 21.74; *p* = < 0.195	χ^2^ = 10.68; *p* = < 0.872	χ^2^ = 20.19; *p* = < 0.264	χ^2^ = 36.76; ***p* = < 0.004**
**Educational Qualification**															
Primary school diploma	0.88 (0.3)	0.11 (0.3)	0.05 (0.2)	0.11 (0.3)	0.05(0.2)	0.88 (0.3)	0.05 (0.2)	0.88 (0.9)	0.29 (0.4)	0.23 (0.4)	0.05 (0.2)	0.29 (0.7)	1.88 (1.7)	0.47 (0.6)	0.58 (1)
Middle school diploma	0.88 (0.6)	0.20 (0.4)	0.12 (0.3)	0.16 (0.3)	0.12 (0.3)	0.80 (0.4)	0.08 (0.2)	0.40 (0.5)	0.36 (0.4)	0.20 (0.4)	0.04 (0.2)	0.76 (0.9)	1.68 (1.4)	3.88 (16.9)	0.44 (0.7)
Secondary school diploma	0.38 (0.4)	0.50 (0.5)	0.20 (0.4)	0.11 (0.3)	0.25 (0.4)	0.75 (0.4)	0.02 (0.1)	0.45 (0.4)	0.38 (0.5)	0.32 (0.6)	0.16 (0.8)	0.42 (0.8)	1.33 (1.4)	0.73 (1)	0.55 (0.7)
Post-secondary degree	0.34 (0.4)	0.24 (0.4)	0.53 (0.5)	0.06 (0.2)	0.57 (0.5)	0.51 (0.5)	0.02 (0.1)	0.53 (0.5)	0.63 (0.6)	0.30 (0.4)	0.38 (1)	0.22 (0.5)	1.44 (1.4)	0.48 (8)	0.51 (0.6)
**Kruskal Wallis Test**	χ^2^ = 27.80; ***p* = < 0.001**	χ^2^ = 15.68; ***p* = < 0.001**	χ^2^ = 24.43; ***p* = < 0.001**	χ^2^ = 1.90; *p* = < 0.594	χ^2^ = 25.95; ***p* = < 0.001**	χ^2^ = 12.96; ***p* = < 0.005**	χ^2^ = 1.96; *p* = < 0.581	χ^2^ = 11.68; ***p* = < 0.009**	χ^2^ = 7.88; ***p* = < 0.048**	χ^2^ = 0.98; *p* = < 0.804	χ^2^ = 11.64; ***p* = < 0.009**	χ^2^ = 10.46; ***p* = < 0.015**	χ^2^ = 2.88; *p* = < 0.409	χ^2^ = 2.38; *p* = < 0.496	χ^2^ = 0.94; *p* = < 0.814
**Profession**															
Employee	0.27 (0.4)	0.31 (0.4)	0.52 (0.5)	0.08 (0.2)	0.49 (0.5)	0.52 (0.5)	0.01 (0.1)	0.57 (0.5)	0.45 (0.6)	0.36 (0.5)	0.45 (1)	0.24 (0.6)	1.04 (1)	0.54 (0.9)	0.42 (0.5)
Freelance worker	0.29 (0.4)	0.58 (0.5)	0.23 (0.4)	0.05 (0.2)	0.23 (0.4)	0.76 (0.4)	0.05 (0.2)	0.64 (0.4)	0.41 (0.5)	0.41 (0.6)	0.05 (0.2)	0.29 (0.5)	1.35 (1.2)	0.35 (0.6)	0.52 (0.7)
Manager	0.75 (0.5)	0.25 (0.5)	0.25 (0.5)	0.25 (0.5)	0 (0)	1 (0)	0 (0)	1 (0)	0.75 (0.5)	0.50 (0.5)	0 (0)	0 (0)	1.75 (1.7)	1 (0.8)	0.50 (0.5)
Temporary collaborator	0.22 (0.4)	0.66 (0.5)	0.22 (0.4)	0 (0)	0.33 (0.5)	0.77 (0.4)	0.11 (0.3)	0.77 (0.4)	0.33 (0.5)	0.33 (0.5)	0 (0)	0.44 (0.7)	1.22 (1.6)	0.44 (0.7)	0.77 (1)
Unemployed	0.51 (0.5)	0.44 (0.5)	0.17 (0.3)	0.13 (0.3)	0.20 (0.4)	0.82 (0.3)	0.03 (0.1)	0.75 (0.4)	0.37 (0.5)	0.10 (0.4)	0 (0)	0.37 (0.7)	1.75 (1.7)	3.79 (15.6)	0.72 (0.9)
Student	1 (0.4)	0.08 (0.2)	0 (0)	0.11 (0.3)	0.16 (0.3)	0.80 (0.4)	0.05 (0.2)	0.55 (0.7)	0.47 (0.5)	0.22 (0.4)	0.05 (0.2)	0.69 (0.9)	2.16 (1.6)	0.55 (0.8)	0.47 (0.8)
Pensioner	1 (0)	0 (0)	0 (0)	0.33 (0.5)	0 (0)	1 (0)	0 (0)	1 (0)	0.33 (0.5)	0.33 (0.5)	0.33 (0.5)	1.33 (1.5)	1 (1)	0.33 (0.5)	0.33 (0.5)
**Kruskal Wallis Test**	χ^2^ = 50.61; ***p* = < 0.001**	χ^2^ = 23.12; ***p* = < 0.001**	χ^2^ = 35.29; ***p* = < 0.001**	χ^2^ = 4.81; *p* = < 0.568	χ^2^ = 18.87; ***p* = < 0.007**	χ^2^ = 16.76; ***p* = < 0.010**	χ^2^ = 2.88; *p* = < 0.823	χ^2^ = 10.36; *p* = < 0.110	χ^2^ = 2.76; *p* = < 0.838	χ^2^ = 8.46; *p* = < 0.206	χ^2^ = 13.89; ***p* = < 0.031**	χ^2^ = 13.88; ***p* = < 0.031**	χ^2^ = 12.73; ***p* = < 0.047**	χ^2^ = 8.09; *p* = < 0.231	χ^2^ = 2.16; *p* = < 0.904
**Marital status**															
Unmarried	0.75 (0.6)	0.09 (0.2)	0.21 (0.4)	0.06 (0.2)	0.27 (0.4)	0.60 (0.4)	0.06 (0.2)	0.36 (0.4)	0.63 (0.6)	0.24 (0.4)	0.42 (1)	0.60 (0.8)	1.90 (1.7)	2.90 (14.7)	0.48 (0.9)
Bachelor	1 (0)	0.05 (0.2)	0.05 (0.2)	0.15 (0.3)	0.25 (0.4)	0.80 (0.4)	0.10 (0.3)	0.70 (0.9)	0.40 (0.5)	0.25 (0.4)	0 (0)	0.70 (0.9)	1.75 (1.4)	0.65 (0.9)	0.60 (0.7)
Married	0.30 (0.4)	0.43 (0.4)	0.36 (0.4)	0.10 (0.3)	0.32 (0.4)	0.69 (0.4)	0.01 (0.1)	0.78 (0.4)	0.31 (0.5)	0.30 (0.5)	0.21 (0.8)	0.23 (0.6)	1.32 (1.3)	0.46 (0.7)	0.44 (0.5)
Divorcee	0.37 (0.4)	0.51 (0.5)	0.27 (0.4)	0.10 (0.3)	0.34 (0.4)	0.75 (0.4)	0.03 (0.1)	0.79 (0.4)	0.58 (0.5)	0.31 (0.5)	0.06 (0.2)	0.37 (0.8)	1.20 (1.4)	1.20 (1.1)	0.75 (0.8)
Widower	1 (0)	0.50 (0.7)	0 (0)	0.50 (0.7)	0 (0)	0.50 (0.7)	0 (0)	1 (0)	0.50 (0.5)	0.50 (0.7)	0 (0)	0.50 (0.7)	1.5 (0.7)	0 (0)	0 (0)
**Kruskal Wallis Test**	χ^2^ = 40.45; ***p* = < 0.001**	χ^2^ = 24.01; ***p* = < 0.001**	χ^2^ = 9.75; ***p* = < 0.045**	χ^2^ = 4.36; *p* = < 0.358	χ^2^ = 1.73; *p* = < 0.785	χ^2^ = 3.17; *p* = < 0.52	χ^2^ = 3.96; *p* = < 0.411	χ^2^ = 14.56; ***p* = < 0.006**	χ^2^ = 11.79; ***p* = < 0.019**	χ^2^ = 0.69; *p* = < 0.952	χ^2^ = 5.31; *p* = < 0.256	χ^2^ = 11.39; ***p* = < 0.022**	χ^2^ = 5.40; *p* = < 0.249	χ^2^ = 18.29; ***p* = < 0.001**	χ^2^ = 5.50; *p* = < 0.239

**Table 4:** Differences between the indicators of the detected variables (user’s resources, referral, intervention model, professional’s considerations) and the independent variables (age, gender, region of origin, educational qualification, profession, marital status) (Kruskal–Wallis test) (N = 160).

Variables	User’s Resources				Referral			Intervention Model							Professional’s Considerations			
	Cognitive Resources	Relational/Social Resources	Emotional/Motivational/ Spiritual Resources	Resources Coming from training/ Personal paths	External Referral (Regional Services)	Referral within the Lègami/ Legàmi Service (Follow-Up, Groups …)	None	Social Support	Psycholo-Gical Rehabilitation	Educational Rehabilitation	Psycho -Social Rehabilitation	Buffering Hypothesis	Psycho-Education	Other Models	Redefining of the Relation-Ship with the Self and with Others	Discom-Fort in the Relation-Ship with the Self and with Others	Showing Hidden Emotions and Expressing Them	Need for Continuous Support
**Age ranges**	Mean (SD)				Mean (SD)			Mean (SD)							Mean (SD)			
10–13	0.77 (0.8)	1 (0.5)	0.22 (0.6)	0 (0)	0.44 (0.5)	0.66 (0.5)	0.22 (0.4)	0.44 (0.5)	0.33 (0.5)	0.11 0.33 (0.3)	0 (0)	0.22 (0.4)	0.22 (0.4)	0 (0)	1.11 (1.3)	0.55 (1)	0 (0)	0.22 (0.4)
14–18	1.20 (1.3)	1 (1)	0.80 (0.8)	0.06 (0.2)	0.40 (0.6)	0.40 (0.5)	0.33 (0.4)	0.20 (0.4)	0.53 (0.5)	0.06 0.33 (0.2)	0 (0)	0.06 (0.2)	0.26 (0.4)	0 (0)	0.8 (0.9)	0.86 (1.4)	0.12 (0.3)	0.26 (0.4)
19–24	1.60 (1.7)	0.90 (0.5)	0.60 (0.9)	0 (0)	0.40 (0.5)	0.50 (0.5)	0.50 (0.9)	0.70 (0.4)	0.40 (0.5)	0 (0)	0.10 (0.3)	0 (0)	0.10 (0.3)	0 (0)	1.3 (1.7)	1.2 (1.5)	0.10 (0.3)	0.40 (0.5)
25–35	0.70 (0.4)	0.95 (0.7)	0.30 (0.5)	0.15 (0.3)	0.45 (0.5)	0.25 (0.4)	0.10 (0.3)	0.45 (0.5)	0.15 (0.3)	0 (0)	0.10 (0.3)	0.10 (0.3)	0.60 (0.5)	0.05 (0.2)	0.75 (0.8)	0.80 (1.7)	0 (0)	0.25 (0.4)
36–46	0.92 (0.8)	0.73 (0.9)	0.60 (0.7)	0.21 (0.4)	0.18 (0.3)	0.20 (0.4)	0.50 (0.5)	0.46 (0.5)	0.23 (0.4)	0.18 (0.3)	0.03 (0.1)	0.13 (0.3)	0.33 (0.4)	0.1 (0.1)	1.24 (1.3)	0.55 (1)	0.10 (0.4)	0.29 (0.5)
47–57	0.48 (0.6)	0.86 (0.9)	0.62 (0.8)	0.03 (0.1)	0.24 (0.4)	0.44 (0.5)	0.31 (0.4)	0.62 (0.4)	0.34 (0.4)	0.17 (0.3)	0.10 (0.3)	0.10 (0.3)	0.13 (0.3)	0 (0)	1 (1)	0.82 (1.5)	0.24 (0.4)	0.37 (0.4)
58–69	0.55 (0.7)	1 (1.3)	0.55 (0.1)	0.11 (0.3)	0.33 (0.5)	0.55 (0.7)	0.33 (0.5)	0.33 (0.5)	0.66 (0.5)	0.11 (0.3)	0 (0)	0.22 (0.4)	0 (0)	0 (0)	1.22 (1.2)	0.33 (0.7)	0.33 (0.5)	0.11 (0.3)
70–85	1.33 (0.5)	0.66 (0.5)	1 (1)	0 (0)	0.33 (0.5)	0.66 (1.1)	0.33 (0.5)	0.66 (0.5)	0.33 (0.5)	0.33 (0.5)	0 (0)	0.33 (0.5)	0.33 (0.5)	0 (0))	0.66 (1.1)	1.33 (1.1)	0 (0)	0.66 (0.5)
**Kruskal Wallis Test**	χ^2^ = 11.12; *p* = < 0.133	χ^2^ = 3.97; *p* = < 0.78	χ^2^ = 9.55; *p* = < 0.78	χ^2^ = 10.70; *p* = < 0.152	χ^2^ = 8.28; *p* = < 0.308	χ^2^ = 14.16; ***p* = < 0.048**	χ^2^ = 12.83; *p* = < 0.076	χ^2^ = 10.28; *p* = < 0.173	χ^2^ = 14.06; ***p* = < 0.050**	χ^2^ = 8.22; *p* = < 0.313	χ^2^ = 5.68; *p* = < 0.577	χ^2^ = 4.95; *p* = < 0.665	χ^2^ = 19; ***p* = < 0.008**	χ^2^ = 3.23; *p* = < 0.861	χ^2^ = 25.36; ***p* = < 0.001**	χ^2^ = 12.50; *p* = < 0.085	χ^2^ = 6.83; *p* = < 0.446	χ^2^ = 3.47; *p* = < 0.838
**Gender**																		
Male	0.70 (1)	0.91 (0.9)	0.47 (0.6)	0.11 (0.3)	0.44 (0.5)	0.44 (0.5)	0.26 (0.6)	0.55 (0.5)	0.32 (0.4)	0.08 (0.2)	0.05 (0.2)	0.17 (0.3)	0.20 (0.4)	0 (0)	1.23 (1.1)	0.70 (1.1)	0.11 (0.3)	0.41 (0.5)
Female	0.90 (0.9)	0.83 (0.8)	0.59 (0.8)	0.12 (0.3)	0.24 (0.4)	0.31 (0.5)	0.40 (0.4)	0.45 (0.4)	0.30 (0.4)	0.14 (0.3)	0.04 (0.2)	0.11 (0.3)	0.30 (0.4)	0.01 (1)	1 (1.2)	0.70 (1.3)	0.12 (0.3)	0.26 (0.4)
**Kruskal Wallis Test**	χ^2^ = 2.74; *p* = < 0.098	χ^2^ = 0.30; *p* = < 0.584	χ^2^ = 0.431; *p* = < 0.584	χ^2^ = 0.21; *p* = < 0.884	χ^2^ = 5.23; ***p* = < 0.022**	χ^2^ = 2.09; *p* = < 0.147	χ^2^ = 4.01; ***p* = < 0.045**	χ^2^ = 1.20; *p* = < 0.272	χ^2^ = 0.04; *p* = < 0.829	χ^2^ = 0.69; *p* = < 0.404	χ^2^ = 0.70; *p* = < 0.791	χ^2^ = 1.03; *p* = < 0.308	χ^2^ = 1.39; *p* = < 0.238	χ^2^ = 0.54; *p* = < 0.461	χ^2^ = 0.37; *p* = < 0.539	χ^2^ = 0.32; *p* = < 0.567	χ^2^ = 1.19; *p* = < 0.275	χ^2^ = 0.86; *p* = < 0.353
**Region of Origin**																		
Lombardia	0.79 (1)	0.70 (0.8)	0.50 (0.6)	0 (0)	0.25 (0.4)	0.41 (0.5)	0.58 (0.7)	0.45 (0.5)	0.50 (0.5)	0.08 (0.2)	0.04 (0.2)	0.08 (0.2)	0.20 (0.4)	0 (0)	1 (1)	1 (1.8)	0.25 (0.5)	0.41 (0.5)
Emilia Romagna	0.60 (0.6)	1 (0.8)	0.20 (0.4)	0.10 (0.3)	0.10 (0.3)	0.40 (0.5)	0.40 (0.5)	0.50 (0.5)	0.30 (0.4)	0 (0)	0.20 (0.4)	0.20 (0.4)	0 (0)	0 (0)	0.90 (1.3)	0.30 (0.6)	0.30 (0.6)	0 (0)
Toscana	1.40 (0.8)	1 (1)	0.60 (0.9)	0 (0)	0.20 (0.4)	0.20 (0.4)	0.40 (0.5)	0.40 (0.5)	0.20 (0.4)	0.20 (0.4)	0.20 (0.4)	0.20 (0.4)	0.40 (0.5)	0 (0)	0.60 (0.5)	1 (1.4)	0.40 (0.5)	0.20 (0.4)
Veneto	0.60 (0.8)	0.40 (0.5)	0.20 (0.4)	0 (0)	0.40 (0.5)	0.20 (0.4)	0.20 (0.4)	0.20 (0.4)	0.40 (0.5)	0 (0)	0 (0)	0 (0)	0.80 (0.4)	0 (0)	1 (1)	1 (1.4)	0.40 (0.5)	0.20 (0.4)
Trentino Alto Adige	0 (0)	0.66 (0.5)	0.33 (0.5)	0 (0)	0.66 (0.5)	0.33 (0.5)	0.33 (0.5)	0 (0)	0.33 (0.5)	0 (0)	0 (0)	0.33 (0.5)	0.33 (0.5)	0 (0)	0.33 (0.5)	1.6 (1.1)	0 (0)	0.66 (1.1)
Friuli Venezia Giulia	0.66 (0.5)	0.66 (1.1)	0.66 (0.5)	0 (0)	0.33 (0.5)	0.33 (0.5)	0 (0)	0 (0)	0 (0)	0 (0)	0 (0)	0 (0)	0.66 (0.5)	0 (0)	0 (0)	3.6 (3.5)	0 (0)	0.33 (0.5)
Piemonte	0.71 (1.1)	0.85 (0.8)	0.28 (0.4)	0.14 (0.3)	0.28 (0.4)	0.42 (0.5)	0.28 (0.4)	0.57 (0.5)	0 (0)	0 (0)	0.14 (0.3)	0 (0)	0.14 (0.3)	0 (0)	1.2 (0.7)	0.14 (0.3)	0 (0)	0.28 (0.4)
Liguria	3 (0)	0 (0)	0 (0)	0 (0)	0 (0)	1 (0)	0 (0)	1 (0)	1 (0)	0 (0)	0 (0)	0 (0)	1 (0)	0 (0)	2 (0)	0 (0)	0 (0)	1 (0)
Umbria	0.80 (0.8)	1.60 (1.5)	0.40 (0.5)	0 (0)	0.20 (0.4)	0.80 (0.4)	0.20 (0.4)	0.120 (0.4)	0.80 (0.8)	0.40 (0.5)	0 (0)	0 (0)	0 (0)	0 (0)	0.80 (1)	0 (0)	0 (0)	0.60 (0.5)
Abruzzo	1 (0)	1 (0)	0 (0)	0 (0)	1 (0)	0 (0)	0 (0)	1 (0)	0 (0)	0 (0)	0 (0)	0 (0)	0 (0)	0 (0)	1 (0)	1 (0)	0 (0)	1 (0)
Lazio	0.92 (1)	0.70 (0.8)	0.69 (0.8)	0.07 (0.2)	0.61 (0.5)	0.23 (0.4)	0.23 (0.4)	0.46 (0.5)	0.30 (0.4)	0.07 (0.2)	0 (0)	0.07 (0.2)	15.3 (0.3)	0 (0)	1.23 (1.9)	0.92 (1.1)	0 (0)	0.38 (0.5)
Marche	1.33 (1.1)	1.66 (2)	2 (2.6)	0.67 (0.5)	0.66 (0.5)	0.33 (0.5)	0 (0)	0.66 (0.5)	0.66 (0.5)	0 (0)	0 (0)	0.33 (0.5)	0 (0)	0 (0)	0 (0)	0.33 (0.5)	0.33 (0.5)	0 (0)
Molise	1 (0)	0 (0)	2 (0)	0 (0)	0 (0)	0 (0)	1 (0)	0 (0)	0 (0)	0 (0)	0 (0)	0 (0)	0 (0)	1 (0)	0 (0)	2 (0)	0 (0)	0 (0)
Campania	0.86 (0.8)	0.73 (0.9)	0.53 (0.8)	0.20 (0.4)	0.66 (0.6)	0.53 (0.6)	0 (0)	0.66 (0.4)	0.26 (0.4)	0.20 (0.4)	0.06 (0.2)	0.13 (0.3)	0.20 (0.4)	0 (0)	1 (1.1)	0.60 (0.9)	0.20 (0.4)	0.53 (0.5)
Puglia	0.88 (0.6)	0.77 (0.8)	0.88 (1)	0 (0)	0.33 (0.5)	0.33 (0.5)	0.33 (0.5)	0.66 (0.5)	0.33 (0.5)	0.22 (0.4)	0.11 (0.3)	0.33 (0.5)	0 (0)	0 (0)	1.55 (0.8)	0.88 (1.1)	0.11 (0.33)	0 (0)
Calabria	0.50 (1)	1.25 (0.9)	0 (0)	0 (0)	0.50 (0.5)	0.25 (0.5)	0 (0)	0 (0)	0.25 (0.5)	0.25 (0.5)	0 (0)	0 (0)	0.50 (0.5)	0 (0)	0.25 (0.5)	0.25 (0.5)	0 (0)	0 (0)
Sicilia	0.95 (1)	0.89 (0.8)	0.67 (0.7)	0.26 (0.4)	0.08 (0.2)	0.26 (0.4)	0.56 (0.5)	0.52 (0.5)	0.26 (0.4)	0.19 (0.4)	0.02 (0.1)	0.13 (0.3)	0.45 (0.5)	0 (0)	1.3 (1.3)	0.52 (1)	0.08 (0.28)	0.28 (0.45)
Sardegna	0.80 (0.4)	0.80 (0.8)	0.40 (0.5)	0 (0)	0 (0)	0.20 (0.4)	0.40 (0.5)	0.40 (0.5)	0 (0)	0 (0)	0 (0)	0.20 (0.4)	0.40 (0.5)	0 (0)	1 (1.2)	0 (0)	0 (0)	0 (0)
**Kruskal Wallis Test**	χ^2^ = 14.66; *p* = < 0.620	χ^2^ = 9.14; *p* = < 0.936	χ^2^ = 18.19; *p* = < 0.377	χ^2^ = 26.19; *p* = < 0.071	χ^2^ = 36.66; ***p* = < 0.004**	χ^2^ = 13.79; *p* = < 0.682	χ^2^ = 29.50; ***p* = < 0.030**	χ^2^ = 19.99; *p* = < 0.274	χ^2^ = 19.52; *p* = < 0.299	χ^2^ = 13.44; *p* = < 0.706	χ^2^ = 12.21; *p* = < 0.787	χ^2^ = 11.44; *p* = < 0.833	χ^2^ = 33.57; ***p* = < 0.010**	χ^2^ = 86.54; ***p* = < 0.001**	χ^2^ = 21.74; *p* = < 0.195	χ^2^ = 10.68; *p* = < 0.872	χ^2^ = 20.19; *p* = < 0.264	χ^2^ = 36.76; ***p* = < 0.004**
**Educational** **Qualification**																		
Primary school diploma	0.82 (0.7)	0.82 (0.5)	0.41 (0.7)	0.05 (0.2)	0.52 (0.5)	0.52 (0.5)	0.17 (0.3)	0.64 (0.4)	0.35 (0.4)	0.11 (0.3)	0 (0)	0.17 (0.3)	0.17 (0.3)	0 (0)	0.94 (1.1)	0.35 (0.7)	0.05 (0.2)	0.35 (0.4)
Middle school diploma	0.96 (1.1)	0.92 (0.9)	0.52 (0.8)	0.08 (0.2)	0.32 (0.5)	0.24 (0.4)	0.36 (0.4)	0.36 (0.4)	0.28 (0.4)	0.04 (0.2)	0 (0)	0.12 (0.3)	0.24 (0.4)	0 (0)	0.68 (0.9)	0.76 (1.2)	0.08 (0.2)	0.16 (0.4)
Secondary school diploma	0.72 (0.8)	1 (0.9)	0.55 (0.9)	0.08 (0.2)	0.30 (0.4)	0.36 (0.5)	0.29 (0.5)	0.42 (0.4)	0.30 (0.4)	0.16 (0.3)	0.07 (0.2)	0.10 (0.3)	0.29 (0.4)	0.01 (0.1)	1 (1.1)	0.85 (1.4)	0.14 (0.3)	0.30 (0.4)
Post-secondary degree	1.04 (0.8)	0.59 (0.7)	0.67 (0.6)	0.22 (0.4)	0.14 (0.3)	0.28 (0.5)	0.57 (0.5)	0.55 (0.5)	0.32 (0.4)	0.14 (0.3)	0.06 (0.2)	0.14 (0.3)	0.32 (0.4)	0.02 (0.1)	1.4 (1.3)	0.59 (1.1)	0.14 (0.4)	0.34 (0.5)
**Kruskal Wallis Test**	χ^2^ = 4.22; *p* = < 0.239	χ^2^ = 5.99; *p* = < 0.112	χ^2^ = 5.24; *p* = < 0.155	χ^2^ = 6.34; *p* = < 0.096	χ^2^ = 9.97; ***p* = < 0.019**	χ^2^ = 4.79; *p* = < 0.188	χ^2^ = 13.94; ***p* = < 0.003**	χ^2^ = 5.08; *p* = < 0.166	χ^2^ = 0.34; *p* = < 0.951	χ^2^ = 2.43; *p* = < 0.487	χ^2^ = 3.09; *p* = < 0.377	χ^2^ = 0.85; *p* = < 0.837	χ^2^ = 1.66; *p* = < 0.644	χ^2^ = 0.79; *p* = < 0.850	χ^2^ = 10.46; ***p* = < 0.015**	χ^2^ = 2.88; *p* = < 0.409	χ^2^ = 2.38; *p* = < 0.496	χ^2^ = 0.94; *p* = < 0.814
**Profession**																		
Employee	0.88 (0.7)	0.72 (0.9)	0.70 (0.8)	0.22 (0.4)	0.16 (0.3)	0.21 (0.4)	0.55 (0.5)	0.50 (0.5)	0.24 (0.4)	0.19 (0.4)	0.04 (0.2)	0.16 (0.3)	0.32 (0.4)	0.03 (0.1)	1.14 (1.1)	0.63 (1)	0.08 (0.2)	0.24 (0.4)
Freelance worker	0.88 (0.9)	0.58 (0.7)	0.52 (0.6)	0.23 (0.4)	0.47 (0.5)	0.41 (0.5)	0.17 (0.3)	0.29 (0.4)	0.35 (0.4)	0.17 (0.3)	0 (0)	0.17 (0.3)	0.29 (0.4)	0 (0)	1 (1.4)	0.52 (0.7)	0.41 (0.6)	0.41 (0.6)
Manager	0.50 (0.5)	1 (0)	1 (1.4)	0 (0)	0 (0)	0.75 (0.5)	0.25 (0.5)	0.50 (0.5)	0.75 (0.5)	0 (0)	0 (0)	0 (0)	0 (0)	0 (0)	1.5 (.5)	0.25 (0.5)	0.50 (0.5)	0.75 (0.5)
Temporary collaborator	0.77 (0.8)	0.55 (0.7)	0.55 (0.7)	0 (0)	0.33 (0.5)	0.33 (0.5)	0.22 (0.4)	0.66 (0.5)	0.22 (0.4)	0.11 (0.3)	0.22 (0.4)	0 (0)	0.22 (0.4)	0 (0)	1 (1.3)	1.2 (2.5)	0 (0)	0.33 (0.5)
Unemployed	0.51 (0.6)	1.20 (1)	0.27 (0.5)	0.03 (0.1)	0.37 (0.4)	0.24 (0.4)	0.34 (0.6)	0.51 (0.5)	0.20 (0.4)	0.10 (0.3)	0.06 (0.2)	0.06 (0.2)	0.34 (0.4)	0 (0)	0.89 (1)	0.62 (1.4)	0.10 (0.4)	0.34 (0.4)
Student	1.16 (0.5)	0.94 (0.7)	0.58 (0.8)	0.02 (0.1)	0.38 (0.5)	0.52 (0.5)	0.25 (0.4)	0.44 (0.5)	0.44 (0.5)	0.05 (0.2)	0.02 (0.1)	0.11 (0.3)	0.22 (0.4)	0 (0)	1.16 (1.2)	0.86 (1.3)	0.08 (0.2)	0.25 (0.4)
Pensioner	0.66 (0.5)	1.33 (0.5)	0 (0)	0 (0)	0 (0)	1 (1)	0.33 (0.5)	0.33 (0.5)	0.66 (0.5)	0 (0)	0 (0)	0.33 (0.5)	0 (0)	0 (0)	1 (1.2)	0.70 (1.2)	0.12 (0.3)	0.20 (0.4)
**Kruskal Wallis Test**	χ^2^ = 7.11; *p* = < 0.311	χ^2^ = 11.32; *p* = < 0.079	χ^2^ = 9.93; *p* = < 0.128	χ^2^ = 15.37; ***p* = < 0.018**	χ^2^ = 12.45; *p* = < 0.053	χ^2^ = 17.53; ***p* = < 0.007**	χ^2^ = 15.80; ***p* = < 0.015**	χ^2^ = 4.38; *p* = < 0.625	χ^2^ = 11.76; *p* = < 0.067	χ^2^ = 5.62; *p* = < 0.466	χ^2^ = 7.38; *p* = < 0.287	χ^2^ = 5.14; *p* = < 0.526	χ^2^ = 4.71; *p* = < 0.581	χ^2^ = 3.23; *p* = < 0.779	χ^2^ = 13.88; ***p* = < 0.031**	χ^2^ = 12.73; ***p* = < 0.047**	χ^2^ = 8.09; *p* = < 0.231	χ^2^ = 2.16; *p* = < 0.904
**Marital status**																		
Unmarried	1 (1.2)	0.81 (0.8)	0.69 (0.9)	0.18 (0.3)	0.24 (0.5)	0.24 (0.4)	0.57 (0.5)	0.42 (0.5)	0.36 (0.4)	0.15 (0.3)	0.06 (0.2)	0.15 (0.3)	0.33 (0.4)	0 (0)	1 (1.2)	0.72 (1.2)	0.09 (0.2)	0.33 (0.4)
Bachelor	1 (1.1)	0.90 (0.8)	0.45 (0.6)	0.10 (0.3)	0.55 (0.5)	0.60 (0.5)	0.20 (0.6)	0.65 (0.4)	0.45 (0.5)	0.05 (0.2)	0.10 (0.3)	0.05 (0.2)	0.15 (0.3)	0 (0)	1.2 (1.3)	0.90 (1.2)	0.05 (0.2)	0.35 (0.4)
Married	0.76 (0.7)	0.81 (0.8)	0.55 (0.6)	0.13 (0.3)	0.27 (0.4)	0.31 (0.4)	0.36 (0.4)	0.42 (0.4)	0.27 (0.4)	0.15 (0.3)	0.05 (0.2)	0.13 (0.3)	0.36 (0.4)	0.01 (0.1)	1.17 (1.2)	0.93 (1.7)	0.17 (0.4)	0.37 (0.6)
Divorcee	0.75 (0.7)	1 (1.1)	0.48 (1)	0.06 (0.2)	0.17 (0.3)	0.37 (0.5)	0.27 (0.4)	0.51 (0.5)	0.27 (0.5)	0.10 (0.3)	0 (0)	0.10 (0.3)	0.13 (0.3)	0.03 (0.1)	0.86 (1)	2 (0)	0.50 (0.7)	0 (0)
Widower	1.5 (0.7)	0 (0)	1.5 (0.7)	0 (0)	0.50 (0.7)	0 (0)	0.50 (0.7)	1 (0)	0 (0)	0 (0)	0 (0)	0.50 (0.7)	0 (0)	0 (0)	1 (1.2)	0.70 (1.2)	0.12 (0.3)	0.30 (0.4)
**Kruskal Wallis Test**	χ^2^ = 2.82; *p* = < 0.588	χ^2^ = 2.75; *p* = < 0.600	χ^2^ = 7.09; *p* = < 0.600	χ^2^ = 2.22; *p* = < 0.695	χ^2^ = 9.71; ***p* = < 0.045**	χ^2^ = 8.62; *p* = < 0.071	χ^2^ = 12.49; ***p* = < 0.014**	χ^2^ = 6.06; *p* = < 0.194	χ^2^ = 3.97; *p* = < 0.410	χ^2^ = 2.23; *p* = < 0.693	χ^2^ = 2.75; *p* = < 0.599	χ^2^ = 3.94; *p* = < 0.414	χ^2^ = 8.53; *p* = < 0.074	χ^2^ = 1.82; *p* = < 0.768	χ^2^ = 11.39; ***p* = < 0.022**	χ^2^ = 5.40; *p* = < 0.249	χ^2^ = 18.29; ***p* = < 0.001**	χ^2^ = 5.50; *p* = < 0.239

## Data Availability

Not applicable.
